# Using Random Forests on Real-World City Data for Urban Planning in a Visual Semantic Decision Support System

**DOI:** 10.3390/s19102266

**Published:** 2019-05-16

**Authors:** Nikolaos Sideris, Georgios Bardis, Athanasios Voulodimos, Georgios Miaoulis, Djamchid Ghazanfarpour

**Affiliations:** 1 Department of Informatics and Computer Engineering, University of West Attica, 12243 Athens, Greece; gbardis@uniwa.gr (G.B.); avoulod@uniwa.gr (A.V.); gmiaoul@uniwa.gr (G.M.); 2 XLIM, University of Limoges, 87060 Limoges CEDEX, France; djamchid.ghazanfarpour@unilim.fr

**Keywords:** random forests, urban planning, machine learning, decision support system

## Abstract

The constantly increasing amount and availability of urban data derived from varying sources leads to an assortment of challenges that include, among others, the consolidation, visualization, and maximal exploitation prospects of the aforementioned data. A preeminent problem affecting urban planning is the appropriate choice of location to host a particular activity (either commercial or common welfare service) or the correct use of an existing building or empty space. In this paper, we propose an approach to address these challenges availed with machine learning techniques. The proposed system combines, fuses, and merges various types of data from different sources, encodes them using a novel semantic model that can capture and utilize both low-level geometric information and higher level semantic information and subsequently feeds them to the random forests classifier, as well as other supervised machine learning models for comparisons. Our experimental evaluation on multiple real-world data sets comparing the performance of several classifiers (including Feedforward Neural Networks, Support Vector Machines, Bag of Decision Trees, k-Nearest Neighbors and Naïve Bayes), indicated the superiority of Random Forests in terms of the examined performance metrics (Accuracy, Specificity, Precision, Recall, F-measure and G-mean).

## 1. Introduction

### 1.1. Description of the Problem

As was formally announced at the meeting of the World Planners Congress and the UN Habitat World Urban Forum in 2008, the part of the total Earth population living in cities is greater than the part that resides in a non-urban environment. In the decade that has elapsed since then, the importance as well as the complexity of urban planning have grown exponentially, yet the related intricacies are not always sufficiently acknowledged. Urban planning is an interdisciplinary process, highly demanding in terms of interconnection among the subsystems involved, as it spreads over a wide range of fields, including legal matters and legislation, political and social issues, capital investment, finance expenditures and others, while being computationally intensive due to the volume of participating data and inherently providing a very limited margin for errors and re-runs.

The availability of an increasing amount of heterogeneous data, stemming from a wide range of sensors installed throughout the city, lately coined as “urban big data”, appears to provide new streams of information to exploit in urban planning. Nevertheless, effectively leveraging such information is far from straightforward, since the involved multidisciplinary stakeholders do not necessarily possess the specialized knowledge and understanding of the concepts from the relevant different domains. Moreover, there is a lack of efficient computational tools that would help translate these massive amounts of data into comprehensible, usable, and even actionable hints in the urban planning and development process.

### 1.2. Urban Planning Challenges

The diverse sources of data that can facilitate urban planning stakeholders, decision makers and other participant actors offer the opportunity to develop and experiment with actual mechanisms for semantic modeling and decision support in realistic operating conditions. This leads to the first aspect of the problem, that of efficient and accessible merging and presentation of several features in an integrated environment. Urban planning requires comprehension of infrastructure and its surrounding environment both in terms of low-level physical characteristics (such as geometric features of buildings and their arrangement), as well as higher-level concepts (e.g., standardization and categorization of buildings and their uses, land use modes, and usage of road networks). 

Α major issue faced by urban planning experts is that of standardization. The need for establishing standards in the field is so immense and suggested by numerous researchers and experts that international organizations have been created for the sole purpose of managing this issue [1,2]. All these data are generated by different sources, encoded in different formats and, usually, not exploitable in their initial form. Furthermore, it is not always possible to convert the data to an exploitable form, while, even when the conversion is feasible, the hazard of data alteration or corruption during conversion is always present. Most often, it is extremely difficult to verify the correctness of the process, partly due to the huge volume of data, since it renders a human-performed visual control practically impossible, but also due to copyright issues. As a result, access to the raw data is not unobstructed, thus greatly impeding the process of verification of the outcome. 

Another issue is the origin of the data with regard to the authority of the provider itself, the validity of the data along with their age. The validity of the data is directly related to the entity that collected or implemented them, the methods and technical means used to collect the data, as well as the amount of time elapsed since collection. It is often the case that there is no information concerning the issues above, but even when it is provided, the data may have been rendered obsolete due to their age. Α city is subject to constant changes, as shops and businesses open and close, roads are converted to bidirectional or unidirectional, new buildings are built and other demolished, to name a few examples.

Since we examine urban data, some of the data sources will originate from open data, which are themselves a subject of study and controversy as their use still presents some predicaments. Most open data providers focus on providing data rather than providing the means to exploit the data, or a system that facilitates data manipulation and queries [3]. Finally, some types of data cannot become publicly available for legal reasons, which may severely impact the exploitability of different, but in some way linked, types of data.

### 1.3. Evolving Technologies and Emerging Applications 

Urban planning is a problem towards the resolution of which, in recent decades, developments in various scientific fields have contributed enormously, resulting among others in software products consequently used for mapping, modeling, storing and analyzing information.

However, the same thing cannot be said for machine learning, one of the most rapidly advancing fields in computer science the last years. The great strides, enabled mainly by the advent of deep learning, have brought about revolutionary changes in a variety of fields, such as computer vision [4,5,6], robotics [7], text analysis [8,9], financial market analysis [10,11], biology [12] and medicine [13], physical sciences e.g., physics [14] and chemistry [15], recommender systems in various domains [16], including tourism and navigation [17,18,19]. On the contrary, the use of artificial intelligence in urban planning and development has been far more limited. State of the art artificial intelligence methods can now be appropriately adapted, fine-tuned, and used to exploit the aforementioned increasingly available heterogeneous “urban big data”. Results of such intelligent data analysis can then be used as input to the decision making process by urban planning stakeholders.

### 1.4. Paper Contribution

In this paper, we present a system that can fuse various types of data from different sources, encode them using a novel semantic model that can capture and utilize both low-level geometric information and higher level semantic information. Among the open data providers and sources, there are public organizations dealing with urban planning (e.g., Estate Property Agency).

One of the main problems affecting urban planning is the appropriate choice of location to host a particular activity (either commercial activity or common welfare service) or the correct use of an existing building or empty space. The most frequently asked questions posed by stakeholders concern finding a suitable site for the construction, for example, of a new school or the construction of a new hospital, while discussion is made on the methods and implementation procedures bearing in mind the public interest [20]. Experts need to take into account a variety of factors, such as population distribution and composition, transport coverage and of course the cost, availability of buildings and spaces, and much more. Similar problems are encountered in finding a fitting site for a specific commercial use (e.g., finding a place suitable to open a restaurant or deciding on the suitability of a particular site). Such problems are the focal point of our work.

In particular, the proposed work is intended to yield the core of a decision support system, which, in turn, dictates the need to maximize the degree of automation. In this paper, the formulated problem, i.e., estimating the suitability of a building or space for a specific use, is treated as a classification problem. We propose the use of random forests classifier, because they tend to be invariant to monotonic transformations of the input variables, and are robust to outlying observations, which are often encountered in the discussed urban data. We also make comparisons through scrutinizing the effectiveness of a wide range of machine learning classifiers, such as Support Vector Machines, FeedForward Neural Networks, Naïve Bayes, and others.

**Figure 1:**
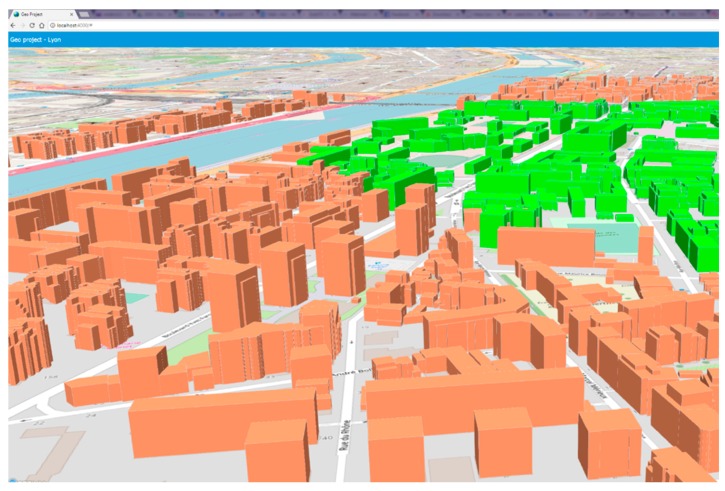
Visualization of a geoquery by the proposed system.

**Figure 2:**
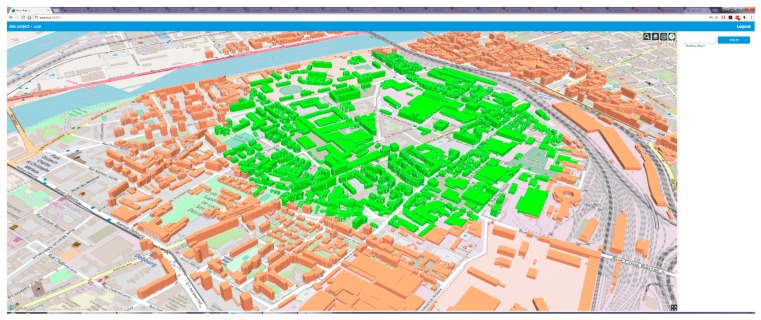
Altered point of view visualization of a geoquery by our system.

In addition to big data management and intelligent decision support, the proposed system also offers a visual interactive environment using current visual techniques (Figure 1 and Figure 2). The inherently large volume of urban data and their type, mostly comprising three-dimensional geometries, makes them practically impossible to conceive in their raw form and strongly suggests their rendering and visualization, a challenging but essential process towards their full exploitation. In problems of such kind, an important factor towards attaining the best possible solution is human intuition and pertinent visualization intensifies human perception facilitating the process at hand.

The aim of the paper is the exploitation of a semantic model of real world urban data that imports, fuses and combines geometric, semantic and routing data, in order to direct this composite and intricate information to machine learning techniques and mechanisms with respect to the pragmatic urban problem of the appropriate choice of location to host a particular activity and compare the results, indicate designated solutions, thus forming the backbone of a decision support system. The results, as well as the urban data are visualized by another component of the system facilitating the Decision Maker.

The remainder of this paper is structured as follows: In Section 2, a review of the related work in machine learning and decision support systems for urban planning problems is provided. In Section 3, we present an overview of the overall system, while Section 4 describes the proposed random forest-based classification mechanism. In Section 5, we experimentally evaluate the proposed method on real-world urban data by comparing it against a variety of classifiers, while Section 6 concludes the paper with a summary of findings. 

## 2. Related Work

A number of research efforts have addressed topics overlapping with the scope of the current work. The latter being multi-faceted, in the sense of employing machine learning, visualization techniques, and geo-spatial databases in a fully functional integrated web environment, suggests presentation of the related work by topic.

### 2.1. Machine Learning for Urban Planning

The work in Reference [21] attempts to apply a machine learning mechanism for large scale evaluation of the qualities of the urban environment. The characteristics used for the learning mechanism vectors are based on the construction and quality of the building façade and the continuity of the street wall as obtained by the relevant street view images using machine vision techniques. The training examples are images labeled by experts and the evaluation results are compared to the public’s opinion of the corresponding buildings, as obtained through an in-situ survey. The authors acknowledge the limited capabilities of the method due to the inherent problems of the source images (perspective, trees, deficiencies imperceptive by machine, etc.) and the possible inconsistency between the experts’ and the public’s evaluations. These problems, enhanced by the high complexity of the problem addressed, are reflected in the results, demonstrating low precision (<50%) and average recall (72%–85%) capability for both observed qualities.

In Reference [22], a procedure to mine points from social networks and feed them to machine learning techniques to estimate aggregated land use is presented. The researchers recognize the problems we debate related to the origin of data and its validity and reliability. However, they deal only with 2d where the mined data consist only of points. The focus is on comparing the results of the machine learning algorithms with those of the census and the ground truth used is another proprietary data set, which does not exclude the existence of errors. The research is macroscopic, performed at a regional level without concentration on city infrastructure and relies on an already available software package (weka) with no additional customization or further development.

In Reference [23], an architecture is proposed to exploit IoT based city sensors dividing each task to low, mid and high level and assigning it to a separate stage of the architecture. The city sensors used include smart home sensors, vehicular networking, weather and water sensors, among others, recording what could be classified as Big Data. The low levels are responsible for data gathering, the intermediate levels perform the task of communications between sensors and framework and the data management and processing, whereas the higher level deals with data interpretation. There is no clear reference to the machine learning mechanisms used, beyond the automatic classification carried out by the ready-made system. In some experiments the data is small (10–15 vehicles). The decision support system is not presented thoroughly and neither has any visualization.

In Reference [24], the urban planning problem of road network expansion and alteration based on existing traffic flow information is addressed. The work infers potentially useful road linkages between city zones that could alleviate traffic flow, and subsequently, improve quality of life and productivity. Alternatively, the load of certain zones participating in high traffic flow yet revealed to be indirectly, and thus, inefficiently connected, may be reduced and transferred to other zones aiming to a more efficient distribution. Data is collected through Bluetooth sensors deployed across the urban area. The proposed model is claimed to also be applicable to new housing, construction, or economic activity, under the limiting condition that these processes will be adequately captured as correlations between zones to guide new routing of the traffic network or load redistribution.

### 2.2. Visualization of Urban Environments

A conceptual framework for urban or regional development design is presented in Reference [25]. The authors’ proposal relies on multifractal modelling in compliance with a number of urban planning principles. A multifractal Sierpinski carpet representing a hierarchical nesting of central places (i.e., urban centres) serves as the theoretical reference model. Fractalopolis GIS-based software [26] is employed to support the application of the concept in relevant case studies. The approach concentrates on the issues of urban development in the sense of expansion/contraction and building density. Moreover, it relies on the presence of relevant data in the form of six shapefiles including buildings (represented by polygons), public transport stations (represented by points), shops and services (represented by points), leisure facilities and green areas (represented by points), non-developable areas (represented by polygons), and the current number of housing units in each local community. The rich semantic content considered as input (e.g., the kind of each service and the frequency of its use) allows for efficient quantitative evaluation of the computed plan and adequate 2D visualization of the plan itself and its efficiency, whereas the approach is synthetic, in the sense that it is producing integrated development plans yet not supporting queries on the efficiency of specific locations and uses.

The work in Reference [27], attempts to visualize the potential sprawling of urban areas. A virtual environment accepts, as input, the geographical orientation and topography as well as the growth of buildings. Urban growth consists of new buildings generated and checked against environmental factors and attractiveness of location, whereas a communal social behavior is programmed to govern the overall building generation. The idea is pertinent to urban planning and relevant decision making and the authors claim similarity of resulting patterns with real urban forms. However, semantic information with respect to buildings is neither exploited nor generated in the process, whereas it is admitted that the rules employed in the virtual urban environment generation mechanism are not derived from actual urban development experience.

The work in Reference [28], concentrates on incorporating semantic information to produce visually appealing 3D models. The latter is achieved by maintaining planar shapes when originally present even in imperfect form, adopting straight building outlines and focusing on detailed building representation while allowing for less detailed surroundings. The semantic content is assigned by previously trained machine learning mechanisms and it is exploited to improve the image recognition and 3D reconstruction process. The achieved accuracy is balanced with a compact and visually appealing 3D reconstruction. The results are also acknowledged to be of decision making interest to certain stakeholders like real-estate agents, however the effort towards any decision support functionality is limited to the care for the aesthetic level of the 3D outcome.

In [29] the emphasis is on the efficiency of the visualization due to the large scale of urban data. Similar to the current work, the visualization is applied on a virtual globe. The work is supportive of a framework for problem solving in urban science presented in Reference [30]. The latter presents a higher level proposal for such a system, integrating and exploiting current capabilities including GIS, heterogeneous data aggregation and efficient visualization. While the proposed framework is wide in scope, the presented implementations of it are limited, not implementing a large part of its functionality. In comparison, the work herein enhances the proposed framework with decision support powered by machine learning techniques while offering an implementation covering the proposed functionality in its entirety.

### 2.3. Semantic Information Exploitation

In the field of semantic exploitation of urban scenes, we have several different approaches. The work of Reference [31] uses multiple sources to reconstruct complete urban environments and enhance the scenes with semantic information. However, in most real scenarios, it is extremely difficult and improbable to acquire or possess that amount of data. Furthermore, as part of evaluation for the proposed method, simulated cities constructed with pseudo-random synthetic data were used. In Reference [32], segmentation mechanisms combining GIS and VHR images is used to semantically classify buildings. However, the lack of appropriate real world samples creates imbalanced data sets which influences the classification results. The categorization of buildings and their variations is limited and constrained in the sense of the amount of the semantic information they administer.

In Reference [33], a method is proposed so that generated meshes from multi-view imagery that present some advantages over LIDAR can be semantically classified. The aforementioned work is formulated in the photogrammetry domain and differs from our proposal in goal and formulation. The work of Reference [34] explains how CityGML works. It aims to describe a whole city, so it is quite extensive, but does not deepen on specific features. The central entity is an abstract building that has geometric characteristics but is not rich in non-geometric information. In addition, there are various levels of accuracy (Lod) that are not affected by the extra information we add. In Reference [35], an interesting description is provided in the part of the process of enriching a model with semantic information. However, it deals with the fragmentation of footprints in buildings and matching them with that existing in databases, which again goes beyond the scope of the current paper.

## 3. Problem Formulation and System Overview

### 3.1. Problem Formulation

To ensure maximum practical value and exploitability, our system is based on the use of real-world open urban data. We have observed that there are numerous open data associated with parking spaces, in the vicinity of the wider geographic area we have selected for our experiment. The suitability of a given space for use as a parking space is a question that meets the requirements of an urban planning problem and additionally has a strong commercial interest. The existence of a tool that can recommend a potential appropriate use of a space or building or make a prediction as to the suitability of an area/building for specific purpose, can be a useful decision support tool for an expert.

Having real-world parking data does not guarantee in itself that we automatically have the knowledge about the salient information therein with regard to the decisive factors that contribute to making a parking lot useful, essential or profitable. In other words, the feature extraction process in this case is far from trivial. In this context, a variety of factors will be explored as potential descriptors, including: distance from landmarks, distance from other parking spots, density of occurrence per specific area, distance from means of public transport along with their plurality in a certain area, distance and density of occurrence with respect to points of touristic interest, and economic and monetary points of interest among others.

### 3.2. System Components

**Figure 3:**
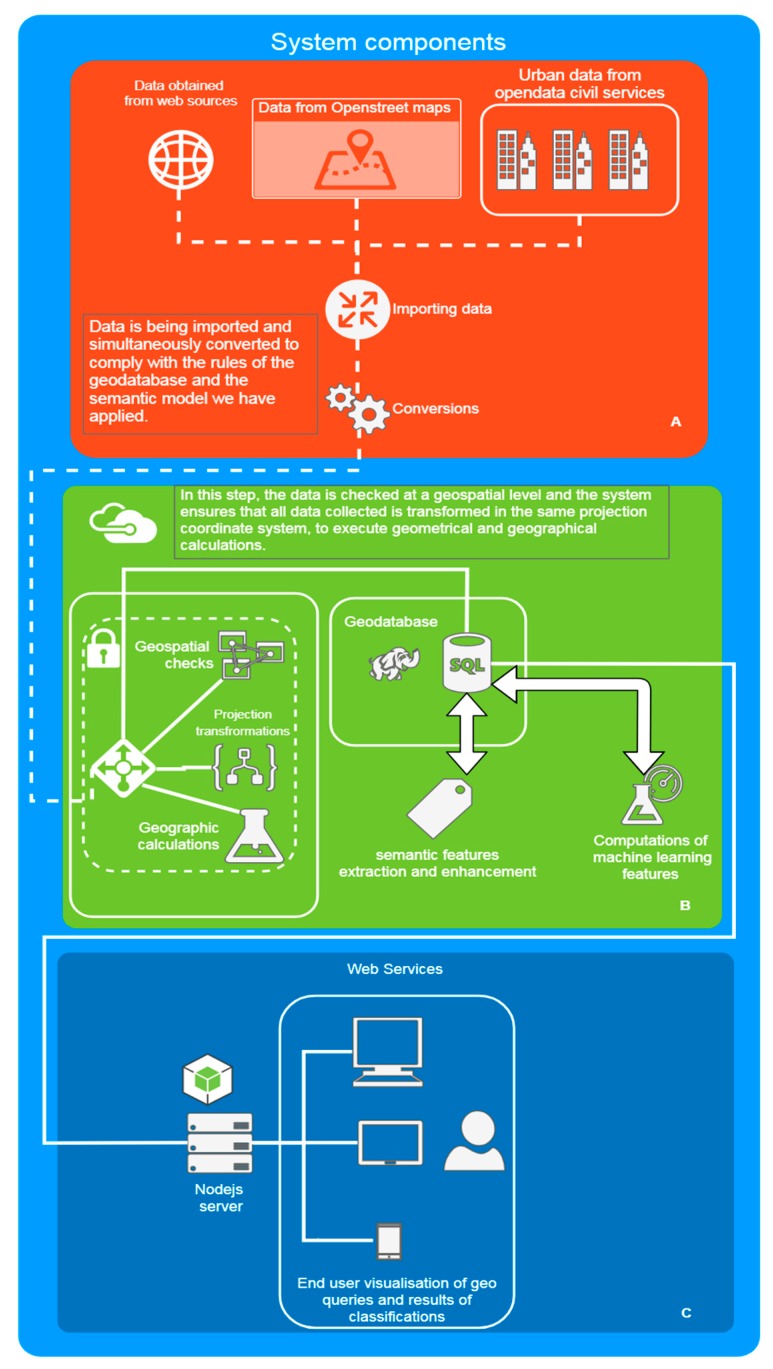
Functional Block Diagram of the proposed System.

The proposed system consists of different components, and operates in distinct stages, as shown in Figure 3. The progression and transition between stages follows sequential procedures for some, while others are being processed in parallel.

The first step is that of data import, following their collection and availability to the system. During their import, the data are appropriately converted to comply with the rules of the geodatabase and the semantic model applied. Relevant checks are also performed at this step, for conversion errors or incomplete data.

In the next step, the data are checked at geospatial level, ensuring that, despite being collected from diverse sources, they are eventually located in the same projection coordinate system, in order to use the same metric system and execute uniform geometrical and geographical calculations.

The third step involves the extraction of semantic features from the data. For example, metadata, use of spaces and buildings, parks and other green areas, public transport stations, where available, are detected and interpreted by our system to populate the ontological model in the geodatabase.

The next stage is data pre-processing and execution of computations for each research question we endeavor to implement, while visualization is performed. For the parking query examined herein, parking spaces and corresponding random buildings are selected, geometric and realistic road distances are calculated, and data is enriched with ontological features extracted from the calculations (e.g., adjacency to specific spaces/buildings based on criteria).

To conclude, the aforementioned data have been imported to our system, combined with corresponding data originating from other open data providers (e.g., the road routable network), undergone the appropriate manipulation to extract the semantic information the framework embassies, while simultaneously calculating the features to be used in the fore coming experiments.

## 4. Random Forests and Other Machine Learning Classifiers for Urban Computing

In this section, we briefly present the random forests classification method, as well as refer to the main classifiers whose suitability in urban planning decision support is examined.

### 4.1. Random Forests

The Random Forests classifier belongs to the broader field of ensemble learning (methods that provide and generate a number of classifiers and aggregate their results). The two best known methods are boosting and bagging of classification trees. The key point of the boosting technique is that consecutive trees add extra weight to the points incorrectly predicted by previous predictors. Upon completion a weighted vote is used for prediction. On the contrary, for bagging (bootstrap aggregating) subsequent trees do not depend on previous trees and each one is independently constructed using a bootstrap sample of the data set. Finally for the prediction a simple majority vote is utilized.

Random Forests provide an extra mantle of randomness to bagging. Apart from the creation process (each tree uses a different bootstrap sample of the data), random forests use different tree construction method. In a standard tree, each node is separated using the best separation score considering all predictor variables. In a random forest, each node is divided using the best score between a subset of the predictors randomly selected on this node. This strategy may initially look odd but it is proven to perform very well compared to many other classifiers, including Support Vector Machines and neural networks. Furthermore, due to that strategy, they are resistant against overfitting [36]. Random forest algorithm consists of three steps:

1. Drawing n bootstrap samples from the original dataset (n refers to the numbers of trees to be constructed).
2. For each of the bootstrap samples, grow a classification tree after modifying it as follows: in each node, rather than choosing the best segregation between all predictors, randomly sample m predictors and select the best separation between them.
3. The predictions for new data can be implemented by aggregating the predictions of the n trees (majority vote).

The appropriate number of trees to be constructed, as well as the number of predictors to be sampled, has also been subjected for studying by many experts, but most researchers including the creator [36] suggest empirical tests according to the dataset characteristics.

According to Reference [37], we can acquire an estimation of the error rate, based on training data, using the following procedure: Firstly, for each iteration using the bootstrap data, predict the data not in the bootstrap sample (Breiman refers to them as “out-of-bag”, or OOB, data) [36] using the tree grown with the original sample. For the second part, we aggregate the OOB predictions. Depending on the implementation of the algorithm, the percentage of times that some data will be out of the bag differs, so we have to aggregate them and then calculate the error rate. This is an indicative way of calculating the error rate called OOB error and generally provides appreciable and accurate estimation of error rate. In the experiments we conducted, we did not use only this error estimation method, but also other metrics (Accuracy, Specificity, Precision, Recall, F_measure and Gmean), to ensure equality and comparability between all classifier experiments.

### 4.2. Support Vector Machines and other Classifiers

Support Vector Machines (SVMs) [38] are discriminative classifiers possessing different construction methods compared to neural networks [39]. The evolution of neural networks preceeded heuristically, with applications and extensive experimentation preceding theory. In contrast, for the development of SVMs, a robust theory was first founded and developed and then followed implementations and experiments.

SVMs can be used for classification or regression. When used for classification, they aspire to find the optimal boundary hyperplane (in the case of two-dimensional data the hyperplane is reduced to a line) that separates the classes. The SVMs construct a hyperplane or a set of hyperplanes in a high or infinite dimension space and aim to select the optimal hyperplane that best separates the points in the input variable space by their class.

The hyperplane that prevails is the one with equal and maximum distance from the closest representatives of each class, namely the support vectors. The distance between the hyperplane and these support vectors creates what is referred as a margin. To calculate the margin the perpendicular distance from the hyperplane to the closest data points is used.

SVMs present significant advantages over Artificial Neural Networks (ANN). They address the drawback frequently observed in ANN, that of suffering from multiple local minima, yielding a solution global and unique. Moreover SVMs have a simple geometric interpretation and their computational complexity does not depend on the dimensionality of the input space. ANNs avail empirical risk minimization, while SVMs on the other hand use structural risk minimization. Finally, SVMs often outperform ANNs in practice because they are less prone to overfitting, which is one of the biggest problem with ANNs. In Section 5, the aforementioned classifiers will be experimentally evaluated regarding their efficacy as an urban planning recommendation mechanism and compared with other machine learning models, including k-Nearest Neighbors and Naïve Bayes.

## 5. Experimental Evaluation

In this Section, we scrutinize the effectiveness of the proposed methods using real-world urban data from the city of Lyon. A series of machine learning techniques have been examined and compared in terms of their efficacy in accurately predicting the suitability of a location/building for a particular use

### 5.1. Urban Data Description and Feature Extraction

Each building, after being successfully imported, is represented in the database by heterogeneous data ranging from concrete geometric properties to semantic information. In particular, for each building identified as unique, the following information is available or may be extracted:

- Dimensions
- Location
- Use
- Material
- Address
- Area
- Height
- Semantic information: use, proximity to other landmarks like media transport stations, places of touristic interest, green areas or rivers, ATM, parking areas
- Media of building (e.g., photos, schematics, contracts).
- Distance to any other building or landmark, both Euclidean or based on shorter route algorithms like Dijkstra

**Figure 4:**
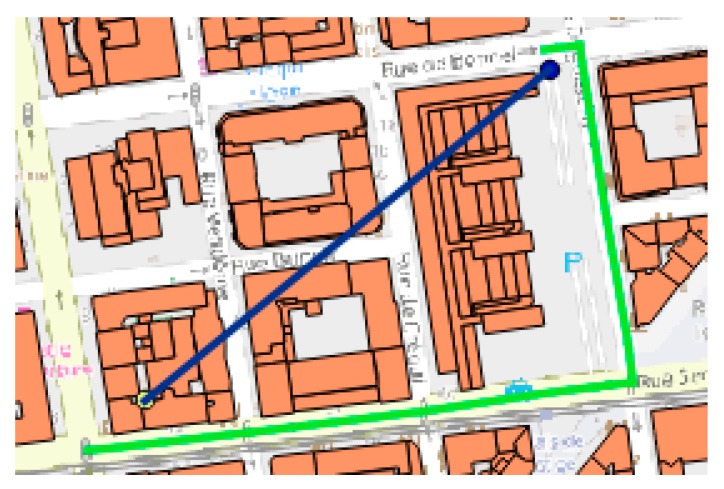
Euclidian distance vs routable road distance.

**Figure 5:**
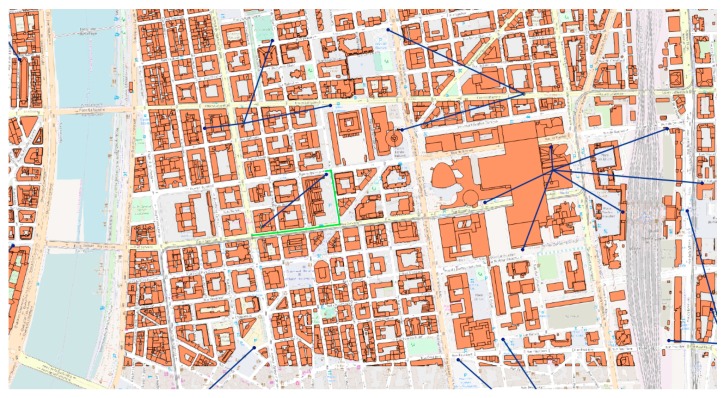
Feature extraction: distance from nearest atm.

The visualization of feature extraction from our system is presented in Figure 4 and Figure 5. In Figure 5 we can observe the discovery of the nearest ATM and subsequently its distance calculation for each point of interest (parking) and its visualizations of the process. In Figure 4, we can see an enlarged section where, in addition to the Euclidian distance, the driving distance, as calculated by our system between the nearest available road access for the involved points of interest is also reflected.

Let the set of buildings:

$$B=\left\{b_{1},b_{2},\ldots ,\;b_{k}\right\},\;k=\mathrm{total}\;\mathrm{number}\;\mathrm{of}\;\mathrm{buildings}\;\mathrm{in}\;\mathrm{database}$$

In order to be able to apply and evaluate the selected machine learning techniques to the current context, we first need to isolate representatives of the two classes that will be the subject of the mechanism’s functionality. In the current work, we focus on the eligibility of a building to be used as a parking service or enterprise. With respect to the need for positive examples, we have used the real-world information contained in the database concerning the actual use of buildings designated as parking lots. Therefore, for the positive examples we have:

(1) P={p∈B,u(p)=parking}

where u(p) indicates the use of the building. Similarly:

(2) N={n∈B,u(n)≠parking}

Evidently: B=P ∪ N

Technically, all buildings in N may be used as negative examples in the current context. However, in order to avoid problems stemming from imbalanced datasets we have chosen to use for the experiments the majority of the members of P as the positive class representatives and we have created different sample data sets of randomly selected non-parking buildings as the negative class representatives. The positive examples correspond to the real parking areas scattered in the vicinity of our area of interest. The recorded real parkings in our dataset are 1000. As a contradiction, the other 1000 buildings, which will constitute an example of negative class, have been randomly selected ensuring obviously they do not belong to the first class. The use of real data gives us the possibility to clearly and directly verify the outcome. We have created eight different negative datasets, the choice of the negative examples being random. The data that will serve as negative examples consists of every other buildings in the dataset since we cannot exclude any of them, due to the lack of a computational model to decide on its suitability.

In particular, for the positive examples and according to the notation above, we have:

(3) $$P_{e}\subset P,\;P_{e}=\left\{p_{1},\;p_{2},\ldots ,p_{1000}\right\}$$

whereas, for the negative examples:

(4) $$N_{e}^{i}\subset N,N_{e}^{i}=\left\{n_{1}^{i},n_{2}^{i},\ldots ,n_{1000}^{i}\right\}$$

$$\overset{8}{\underset{i=1}{\cap }}N_{e}^{i}=\emptyset$$

In order for these datasets to be used in the training and evaluation, each participating building has to be represented by a feature vector. Each element of these vectors represents a metric contributing to the aforementioned processes, whereas the value contained in the feature vector of a building represents the assessment of the real-world data of the building against this metric. In the general case, each feature value is a real number, hence, we may consider a function mapping the building properties, as expressed in the database, to an n-dimensional feature vector:

$$F:B\mapsto {\mathbb{R}}^{n}$$

In practice, each feature vector consists of the following features:

- Number of other parking places in the area (1000 m)
- Distance to the nearest next parking (Euclidean)
- Number of ATMs at a distance of 1000 m
- Distance from the nearest ATM (Euclidean)
- Distance from the nearest ATM (Dijkstra using the actual routable road network)
- Number of spots of tourist interest (1000 m)
- Distance to the nearest spot of tourist interest
- Building area (in m^2^)

As an output of the experiments, a binary classification is desired between ‘parking’ and ‘no parking’ classes. Where the implementations allow it, the same word classes have been used, while in the rest of occasions, where the output has to be scalar, to maintain uniformity 1 corresponds to the ‘parking’ class and 0 to ‘no parking’ class. In the following, and in compliance with the above notation, we will represent by P_c_ the buildings corresponding to the set of samples predicted as positive by the classifier, and by N_c_ the buildings corresponding to the set of samples predicted as negative by the classifier.

### 5.2. Experimental Setup

The lack of a mathematical model to coherently describe the discussed urban data and its characteristics as well as the complexity of the problem make the selection of an appropriate classifier to automatically and successfully predict the suitability of urban locations for particular uses a challenging task.

To ensure a sound and solid experimental evaluation, the tests performed should be expanded and replicated in multiple sample data sets as described previously. To that end we created 8 different sample data sets, each containing features of the 1000 positive examples, i.e., the actual parking areas, which are the same in all sample data sets, and features of 1000 negative examples, i.e., randomly selected buildings, which are different in each sample data set. For each sample data set, two subclasses of experiments have been created: The former uses the entire data and randomly chooses the sections used for training, validation and testing using a ratio of 85%, 5%, and 10%, respectively, while in the other, we masked a segment of data completely from the classifier, only to present it as input after the phase of training, for testing.

In the following presentation of the assessment of the results, we have adopted the following terms:

- True Positives (TP): The cases in which the classifier predicted yes and the actual sample’s class was also yes, formally $TP\;=\;P_{c}\;\cap \;P_{e}$
- True Negatives (TN): The cases in which the classifier predicted no and the actual sample’s class was no, formally $TN\;=\;N_{c}\;\cap \;N_{e}^{i}$
- False Positives (FP): The cases in which the classifier predicted yes and the actual sample’s class was no, formally $FP\;=\;P_{c}\;\cap \;N_{e}^{i}$
- False Negatives (FN): The cases in which the classifier predicted no and the actual sample’s class was yes, formally $FN\;=\;N_{c}\;\cap \;P_{e}$

The above are summarized in the following table (Table 1):

For the evaluation of the results, the following metrics are used:

- Accuracy: the ratio of the number of correct predictions to the total number of input samples.
  $$Accuracy=\;\frac{Number\;of\;correct\;predictions}{Total\;number\;of\;predictions\;made\;}=\frac{\left|TP\right|+\left|TN\right|}{\left|P_{c}\right|+\left|N_{c}\right|}$$
- Specificity: It corresponds to the proportion of negative samples that are mistakenly considered as positive, with respect to all negative samples.
  $$Specificity=\;\frac{False\;Positive}{False\;Positive+\;True\;Negative}=\frac{\left|FP\right|}{\left|N_{e}^{i}\right|}$$
- Precision: It is the number of correctly predicted positive results divided by the number of all samples predicted as positive by the classifier.
  $$Precision=\;\frac{True\;Positive}{\;True\;Positive+False\;Positive}=\frac{\left|TP\right|}{\left|P_{c}\right|}$$
- Recall (or Sensitivity): It is the number of correctly predicted positive results divided by the number of all positive samples regardless of prediction (all samples that should have been identified as positive).
  $$Recall=\;\frac{True\;Positive}{\;True\;Positive+False\;Negative}=\frac{\left|TP\right|}{\left|P_{e}\right|}$$
- F1 measure: is the Harmonic Mean between Precision and Recall. Its range is [0, 1]. It provides information on how precise the classifier is (how many instances it classifies correctly), as well as how robust it is (if it misses a significant number of instances).
  $$F1=\;2\frac{1}{\;\frac{1}{Precision}+\frac{1}{Recall}}$$
- G-Mean: The geometric mean (G-Mean) is the root of the product of class-wise sensitivity. This measure tries to maximize the accuracy on each of the classes while keeping these accuracies balanced. For binary classification G-mean is the squared root of the product of the sensitivity and specificity.
  $$G-Mean=\;\sqrt{Sensitivity\cdot Specificity}$$

### 5.3. Experimental Results

#### 5.3.1. Experiment Description

Given the fact that the problem of predicting the appropriateness of city locations for specific uses using real-world urban data and machine learning has not, to our knowledge, been studied before in the literature, it was deemed useful and necessary to conduct a detailed experimentation process considering a variety of machine learning classification methods, the results of which are compared and discussed. The examined classifiers include: feedforward artificial neural networks (multilayer perceptrons), Support Vector Machines, bag of decision trees and random forests, k-Nearest Neighbors and Naïve Bayes. In the subsections that follow, a detailed presentation of the experimental results for each method is provided, followed by a comparative analysis.

For the classifiers, we also need to assess the margin for augmenting their performance through optimization of their parameters. The results we quote are the ones subsequent to the several stages of optimization. For most we followed a technique often used in machine learning lately, called Bayesian Optimization. In machine learning problems dealing with several hyperparameters, the process to tune the classifier usually frequently involves costly plentiful costly evaluations both in computational resources and time. To avoid that we could use Bayesian Optimization to optimize the parameters. We can build a probabilistic model for the objective and compute the posterior predictive distribution integrating all the possible true functions, thus leading to optimizing a cheap proxy function instead whose model is much cheaper than the true objective. The main insight of the idea is to make the proxy function exploit uncertainty to balance exploration against exploitation. However, this solution is not universal, nor yielding the best results in all occasions, especially in neural networks [40], where manual optimization was applied.

#### 5.3.2. Multilayer Perceptron Results

**Figure 6:**
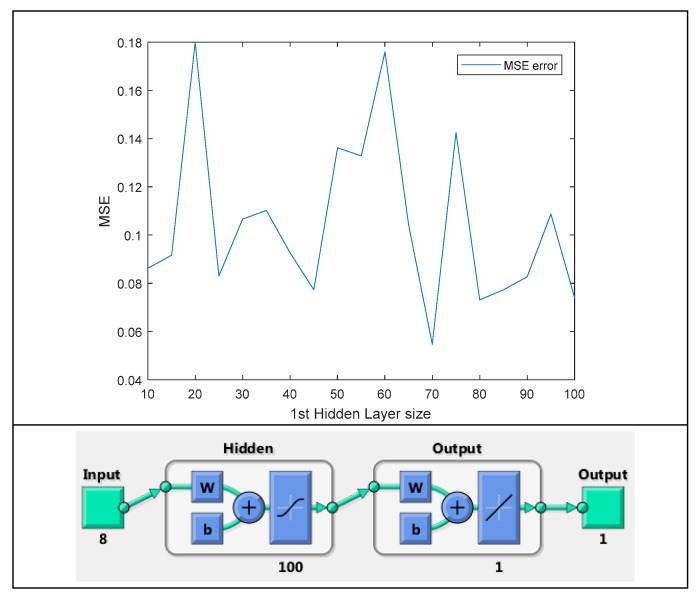
Architecture and graph of Mean Square Error (MSE) plot as varied for 1st layer number of neurons.

The first set of experiments involves the optimization of neural networks, i.e., multilayer perceptrons (MLP), specifically the network architecture, and the multitude of hidden layers as well as neurons per layer. We will initially test two-layer architectures by keeping the number of neurons in the 2nd layer stable and perform testing for the number of neurons in the first layer (Figure 6), since it is often argued that problems rarely need to use over 2 hidden layers of neurons. We conclude that minimal error occurs for 70 neurons. Performing tests respectively for the second layer differentiate results to a negligible degree, so the optimal solution is to keep 15 neurons in the 2nd layer, which is a good trade-off of complexity over results and time. Tests were also performed with single layer perceptron but did not produce near as promising results. As a metric of performance, the Mean Square Error (MSE) of misclassification was used.

Subsequently the configuration providing the best result was chosen for the continuation of experiments and analysis of behavior in unknown inputs where the first results were not encouraging, since there were data pockets for which the Mean Square Error of misclassification ranged from 32%–36%, which differs greatly from network performance in the previous experiment as shown in Table 2. There are numerous other parameters in the architecture of the network (hidden neuron activation functions, seasons, learning rate, etc.) we customized and performed further testing, but we still encountered networks’ usual problems of local minima and overfitting that prevented achieving better results.

#### 5.3.3. Support Vector Machines (SVM)

The initial tests were carried out using the default radial basis function.

$$RBF\;\left(Gaussian\right)\;kernel\;K\left(x_{i},x_{j}\right)=\;e^{-\frac{{\left|\left|x_{i}-x_{j}\right|\right|}^{2}}{2\sigma^{2}}}$$

For the evaluation of the results, in addition to the MSE used previously, SVM supports the kfoldloss metric that gives more valid results as it does not randomly select a percentage of the inputs to hide it and use it for control but separates inputs into random parts of a particular size and tests them all at the testing stage. So all the data, at some time, will be used as both training input and testing input.

Using MSE to evaluate the results, the error ranges at very low levels (around 7-8%), but with the stricter and more accurate kfoldloss the misclassification varies in the range of 18-21%. In combination with our observation from last experiment, we tend to assume the SVM is a better suited classifier for our case than MLP.

Proceeding with the next stage of experiment we intentionally hide some input data and ask the SVM to classify the hidden inputs. The results approximately present the same error percentages as a kfoldloss metric.

Interestingly, in separate experiments on positive and negative inputs (that is, if we only give vectors of parking areas as input and then only non-parking we observe that it has a very high efficiency (>92%) predicting correctly the negative class, that is, we can present it to building and can say with great efficiency that it is not a parking.

Deriving from the latter deduction it became prominently perceptible that it could prove propitious examining alternate configurations in the SVM architecture (kernels and their hyperparameters), using the aforementioned technique Bayesian Optimization.

**Figure 7:**
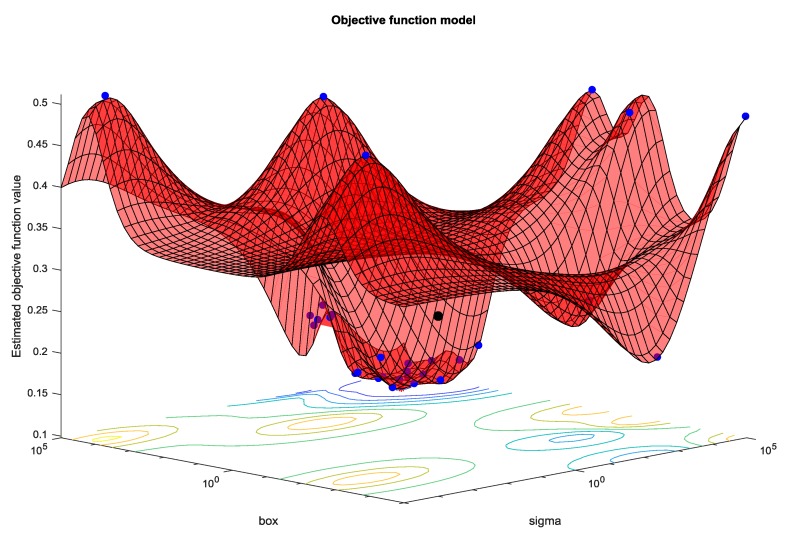
Optimization of hyperparameters box and sigma.

The hyperparameters we try to optimize in this case, are box and sigma (Figure 7). Box refers to the slack variable which controls the error margin we allow the classifier to undergo. Even though we want our classifier to make no mistakes at all and find a hyperplane that separates all positive and negative examples without exemption, sometimes it is preferable to allow some mistakes, because absolutely strict separation can lead to poorly fit models. In the aforementioned cases of complex non-linearly separable real word problems some examples can also be mislabeled or extremely unusual (noise in the data). In order to achieve a better overall solution or a solution at all in other cases, we must allow some misclassified points, and our goal is altered to discovering the solution containing the least mistakes.

By using the optimized parameters in the unknown data, we perceive a much better performance, in random samples the MSE is limited to 8–9% while the kfoldloss (for every possible combination of the new hidden inputs) varies about 16%–18% (Table 3).

#### 5.3.4. Bag of Decision Trees & Random Forests

Tree-based methods partition the feature space into a set of rectangles, and then fit a simple model in each one. Plain decision trees (either classification or regression) present drawbacks and limitations: They have a very high tendency to overfit the data, small variations in the data might result in a completely different tree providing us with unstable results, their implementations are based on heuristic algorithms such as the greedy algorithm where locally optimal decisions are made at each node. Such algorithms cannot guarantee to return the globally optimal decision tree.

**Figure 8:**
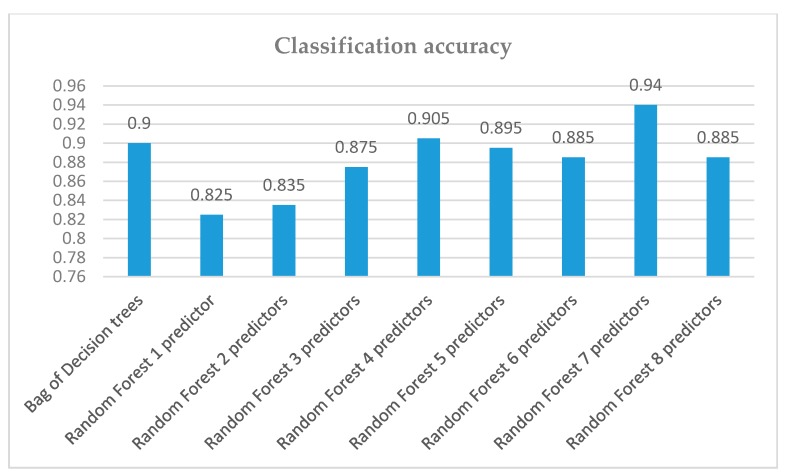
Behavior of Random Forest classifier with different number of features.

Therefore several other strategies have been adopted to confront the above such as pruning and bootstrap aggregation (bagging). In test conducted, it proved to be a very promising classifier with a misclassification error that does not exceed 8%–10% (Figure 8).

Those results motivated the employment of the random forests classifier to our data which provides an improvement over bagged trees by way of a random small tweak that decorrelates the trees. Unlike bagging, in the case of random forests, as each tree is constructed, only a random sample of predictors is taken before each node is split. Since at the core, random forests too are bagged trees, they lead to reduction in variance. Experimenting with the number of predictors, we advance to even better results and classification success, at times reaching 96% (Table 4).

#### 5.3.5. K Nearest Neighbors (KNN)

K nearest neighbors algorithm may be one of the simplest classification algorithms but produces generally competitive results. It is non-parametric which means it does not presume in advance any models for the data distribution and its explicit training phase is minimal, making it very fast. However this also means that most data is needed during the test phase so altering the training data may conclude to substantial variations of the results or poor classification models. The mean error from all the data sets used ranges mostly around 30% (Table 5).

#### 5.3.6. Naive Bayes

Naïve Bayes method is based on the Bayes’ theorem that provides a way to calculate posterior probability. It assigns the most likely class to a given example described by its feature vector, based on the naïve assumption that features are independent given class, that is, $P\left(X|C\right)=\prod_{i=1}^{n}P\left(X_{i}|C\right)$ where $X=\left(X_{1},\ldots ,\;X_{n}\right)$ is a feature vector and C is a class. The predictors are assumed conditionally independent. Even though this assumption is unrealistic and often violated in practice [41], the classifier is remarkably successful and tends to yield results comparable with those attained by far more sophisticated techniques.

It is a fast algorithm performing well in multiclass scenarios, but in our dataset test case, its drawbacks prevailed and it presented poor performance compared to other classifiers and the error ranges from 25–27% in the hidden data test (Table 6).

### 5.4. Comparisons–Discussion

The results of the comprehensive comparisons can be aggregated in the following table and graphs. In Table 7, the average of the metrics applied for all datasets of each classifier after its optimization is presented.

**Figure 9:**
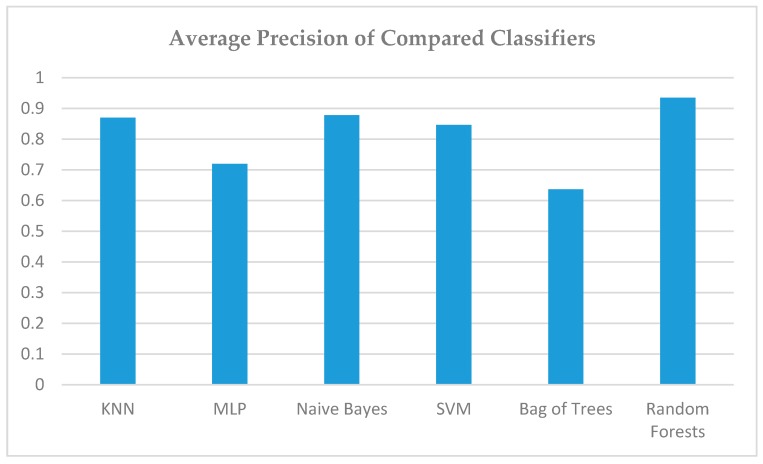
Comparison of Precision of all Classifiers.

In Figure 9, the results of accuracy of compared classifiers are presented. We observe that the use of the proposed machine learning techniques with proper parameterization and training can provide decision support input towards appropriate solutions in urban planning problems.

**Figure 10:**
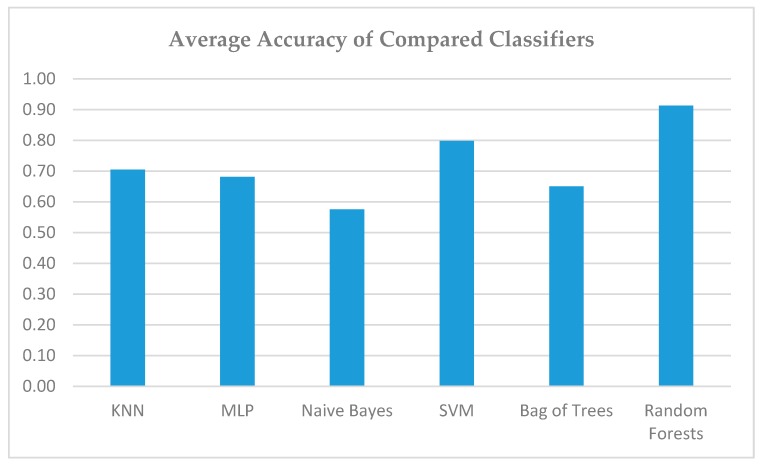
Comparison of Accuracy of all Classifiers.

Our reasoning that the large volume and diversity of data combined with the absence of a computational model is an ideal field of action for the aforementioned techniques was confirmed. In Figure 10, we present the average precision of all classifiers. We observe that apart from accuracy, random forests also prevail in the specific metric with high success rates, i.e., effectiveness in correctly identifying the real parking lots among a set of random buildings and spaces with very few false positives.

**Figure 11:**
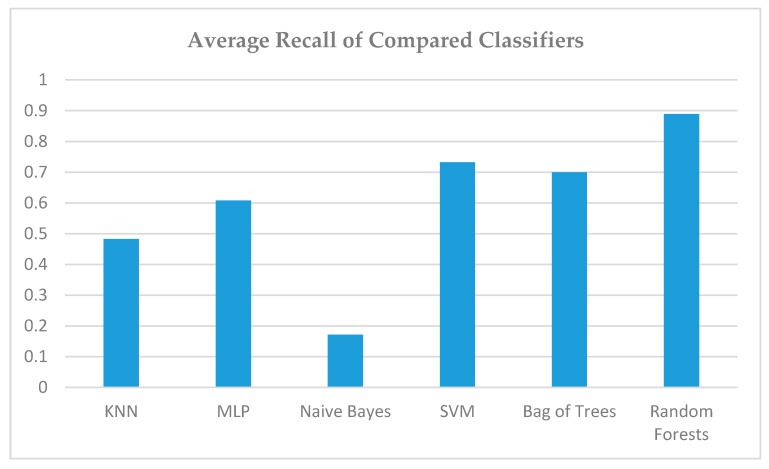
Comparison of Recall of all Classifiers.

In Figure 11, the recall metric is presented, where one can notice the divergence in performance between the predominant (random forests) and the second best approach (SVM), as the latter presented several more false negatives (classified real parking lots as non-suitable for parking use). Random forests, on the other hand, yield consistently good rates in terms of both precision and recall.

**Figure 12:**
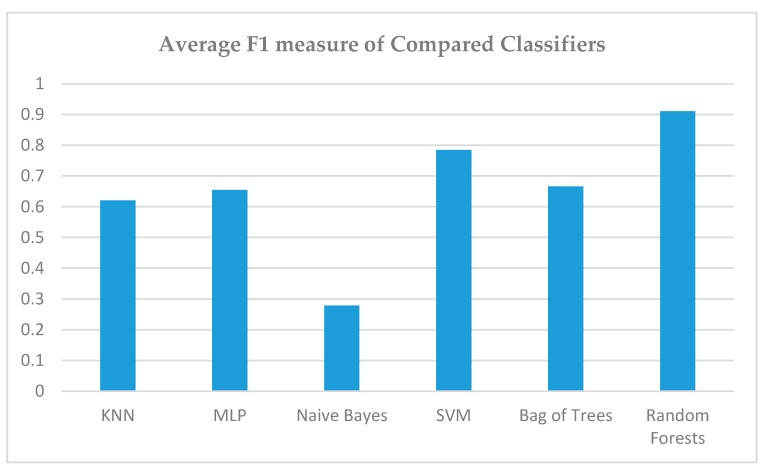
Comparison of F1 measure of all Classifiers.

Finally, in Figure 12, the F1 measure does not present significant differences from the aforementioned conclusions. It is worthwile commenting on the consistently lower performance in all the metrics by the other classifiers. Only SVMs have achieved comparable, but inferior results.

Particularly in the specific problem that we cited concerning the ability to reach a decision for a proposed use of a site, based on given data that may be incomplete and without the intervention of an expert, we find that we have positive results which in exceptional cases yield very high classification scores (even 96% successful classification for some datasets).

## 6. Conclusions

In this work, we presented a visual semantic decision support system that can be used in the context of urban planning applications. One of the main problems affecting urban planning is the appropriate choice of location to host a particular activity (either commercial activity or common welfare service) or the correct use of an existing building or empty space.

The proposed system fuses and merges various types of data modalities from different sources of urban data, encodes them using a semantic model that can capture and utilize both low-level geometric information and higher level semantic information. For feature extraction, a variety of factors have been explored as potential descriptors, including: Attributes of the actual building or location, distance from landmarks, distance from specific similar spots, density of occurrence per specific area, distance from means of public transport along with their plurality in a certain area, distance and density of occurrence with respect to points of touristic interest, economic, and monetary points of interest among others. In the sequel, the system employs a machine learning model based on random forests to estimate the suitability of different city spaces for specific urban uses. The proposed methods have been validated on real-world data and compared with a wide range of machine learning techniques, such as K-nearest neighbors, Naïve Bayes, SVM, and others, and the evaluation indicates promising results.

As future work directions, we intend to acquire and add more urban data, such as traffic data for more realistic distance calculations, and parking revenue data and parking traffic to examine that aspect as well. In addition, we are exploring potential ways of transforming and preparing the data to feed it into convolutional neural network and deep learning and evaluate and compare results. Finally, we aim at replicating the experiment for other cities, provided that we can acquire similar real-world data.

## Figures and Tables

**Table 1:** Summary of prediction results.

		Actual Class	
		YES ($P_{e}$)	NO ($N_{e}^{i}$)
**Classifier’s Prediction**	YES ($P_{c})$	TP	FP
	NO ($N_{c})$	FN	TN

**Table 2:** Results for Artificial Neural Networks.

Dataset	Accuracy	Specificity	Precision	Recall	F1 Measure	G-Mean
1	0.790	0.84	0.82	0.74	0.78	0.79
2	0.740	0.92	0.88	0.56	0.68	0.72
3	0.660	0.72	0.68	0.60	0.64	0.66
4	0.640	0.67	0.65	0.61	0.63	0.64
5	0.640	0.68	0.65	0.60	0.63	0.64
6	0.630	0.78	0.69	0.48	0.56	0.61
7	0.675	0.65	0.67	0.70	0.68	0.67
8	0.675	0.78	0.72	0.57	0.64	0.67

**Table 3:** Results for SVMs.

Dataset	Accuracy	Specificity	Precision	Recall	F1 Measure	G-Mean
1	0.85	0.91	0.90	0.78	0.83	0.84
2	0.85	0.90	0.89	0.80	0.84	0.85
3	0.77	0.81	0.79	0.73	0.76	0.77
4	0.77	0.80	0.78	0.73	0.76	0.76
5	0.78	0.87	0.84	0.69	0.76	0.77
6	0.80	0.90	0.88	0.70	0.78	0.79
7	0.78	0.83	0.81	0.73	0.77	0.78
8	0.80	0.90	0.88	0.70	0.78	0.79

**Table 4:** Results for optimized Random Forests.

Dataset	Accuracy	Specificity	Precision	Recall	F1 Measure	G-Mean
1	0.96	0.98	0.98	0.93	0.95	0.95
2	0.94	0.98	0.98	0.89	0.93	0.93
3	0.86	0.93	0.92	0.79	0.85	0.86
4	0.92	0.93	0.93	0.91	0.92	0.92
5	0.93	0.92	0.92	0.94	0.93	0.93
6	0.93	0.92	0.92	0.93	0.93	0.92
7	0.92	0.92	0.92	0.92	0.92	0.92
8	0.86	0.92	0.91	0.80	0.85	0.86

**Table 5:** Results of KNN.

Dataset	Accuracy	Specificity	Precision	Recall	F1 Measure	G-Mean
1	0.73	0.92	0.87	0.53	0.66	0.70
2	0,71	0.94	0.89	0.48	0.62	0.67
3	0.72	0.90	0.84	0.53	0.65	0.69
4	0.69	0.92	0.85	0.46	0.60	0.65
5	0.71	0.92	0.86	0.50	0.63	0.68
6	0.68	0.91	0.83	0.44	0.58	0.63
7	0.68	0.93	0.86	0.43	0.57	0.63
8	0.69	0.87	0.80	0.51	0.62	0.67

**Table 6:** Results of Naïve Bayes.

Dataset	Accuracy	Specificity	Precision	Recall	F1 Measure	G-Mean
1	0.77	0.85	0.82	0.68	0.74	0.76
2	0.74	0.85	0.81	0.63	0.71	0.73
3	0.73	0.78	0.76	0.68	0.72	0.73
4	0.73	0.78	0.76	0.68	0.72	0.73
5	0.73	0.78	0.76	0.68	0.72	0.73
6	0.73	0.78	0.76	0.68	0.72	0.73
7	0.73	0.78	0.76	0.68	0.72	0.73
8	0.73	0.78	0.76	0.68	0.72	0.73

**Table 7:** Average metrics for all datasets of all classifiers.

Dataset	Accuracy	Specificity	Precision	Recall	F1 Measure	G-Mean
MLP	0.681	0.755	0.719	0.608	0.655	0.674
SVM	0.799	0.865	0.846	0.733	0.784	0.796
KNN	0.699	0.914	0.850	0.485	0.617	0.665
Naive Bayes	0.736	0.798	0.770	0.674	0.718	0.733
Bag of Decision trees	0.651	0.601	0.636	0.700	0,666	0.649
Random Forest	0.913	0.938	0.934	0.889	0.910	0.912
